# Glucoregulatory and Anti-Inflammatory Activities of Peptide Fractions Separated by Electrodialysis with Ultrafiltration Membranes from Salmon Protein Hydrolysate and Identification of Four Novel Glucoregulatory Peptides

**DOI:** 10.3390/membranes11070528

**Published:** 2021-07-14

**Authors:** Loïc Henaux, Karina Danielle Pereira, Jacinthe Thibodeau, Geneviève Pilon, Tom Gill, André Marette, Laurent Bazinet

**Affiliations:** 1 Department of Food Sciences and Laboratory of Food Processing and Electromembrane Processes (LTAPEM), Université Laval, Quebec City, QC G1V 0A6, Canada; loic.henaux.1@ulaval.ca (L.H.); jacinthe.thibodeau.1@ulaval.ca (J.T.); 2 Institute of Nutrition and Functional Foods (INAF), University Laval, Quebec City, QC G1V 0A6, Canada; genevieve.pilon@criucpq.ulaval.ca (G.P.); andre.marette@criucpq.ulaval.ca (A.M.); 3 Laboratory of Biotechnology, School of Applied Sciences, University of Campinas (UNICAMP), Limeira 13484-350, SP, Brazil; kdp.thomaz@gmail.com; 4 Institute of Biosciences, State University (UNESP), Rio Claro 13506-900, SP, Brazil; 5 Department of Medicine, Faculty of Medicine, Quebec Heart and Lung Institute Cardiology Group, Université Laval, 2725 Chemin Ste-Foy, Quebec City, QC G1V 4G5, Canada; 6 Department of Process Engineering and Applied Science, Dalhousie University, P.O. Box 15000, Halifax, NS B3H 4R2, Canada; Tom.Gill@dal.ca

**Keywords:** electrodialysis with filtration membrane, salmon protein hydrolysate, bioactive peptides, anti-inflammatory activity, glucose uptake, hepatic glucose production

## Abstract

Natural bioactive peptides are suitable candidates for preventing the development of Type 2 diabetes (T2D), by reducing the various risk factors. The aim of this study was to concentrate glucoregulatory and anti-inflammatory peptides, from salmon by-products, by electrodialysis with ultrafiltration membrane (EDUF), and to identify peptides responsible for these bioactivities. Two EDUF configurations (1 and 2) were used to concentrate anionic and cationic peptides, respectively. After EDUF separation, two fractions demonstrated interesting properties: the initial fraction of the EDUF configuration 1 and the final fraction of the EDUF configuration 2 both showed biological activities to (1) increase glucose uptake in L6 muscle cells in insulin condition at 1 ng/mL (by 12% and 21%, respectively), (2) decrease hepatic glucose production in hepatic cells at 1 ng/mL in basal (17% and 16%, respectively), and insulin (25% and 34%, respectively) conditions, and (3) decrease LPS-induced inflammation in macrophages at 1 g/mL (45% and 30%, respectively). More impressive, the initial fraction of the EDUF configuration 1 (45% reduction) showed the same effect as the phenformin at 10 μM (40%), a drug used to treat T2D. Thirteen peptides were identified, chemically synthesized, and tested in-vitro for these three bioactivities. Thus, four new bioactive peptides were identified: IPVE increased glucose uptake by muscle cells, IVDI and IEGTL decreased hepatic glucose production (HGP) of insulin, whereas VAPEEHPTL decreased HGP under both basal condition and in the presence of insulin. To the best of our knowledge, this is the first time that (1) bioactive peptide fractions generated after separation by EDUF were demonstrated to be bioactive on three different criteria; all involved in the T2D, and (2) potential sequences involved in the improvement of glucose uptake and/or in the regulation of HGP were identified from a salmon protein hydrolysate.

## 1. Introduction

Type 2 diabetes (T2D) mellitus is a complex metabolic disorder and progressive disease [1]. T2D is associated with environmental causes and behavioral changes as overeating, smoking, physical inactivity, excessive alcohol, and ”junk” food consumption [2]. According to the World Health Organization (WHO), non-communicable diseases (including T2D) are the world’s biggest killers. Indeed, annually, more than 36 million people die from NCDs (63% of global deaths) [3]. Moreover, according to a recent report from the International Diabetes Federation, in 2019, 463 million of people aged between 20 to 79 years had the T2D, [4]. Nevertheless, in order to prevent NCDs and T2D, promoting healthy diet and physical activity are an integral part of the WHO’s “Global action plan for the prevention and control of non-communicable diseases 2013–2020” [3]. However, a high proportion of people at risk do not follow these recommendations, and the T2D continues to progress [5].

Increased fish consumption has been suggested to improve the metabolic syndrome (MetS), to reduce the incidence of T2D, and to protect against cardiovascular disease (CVD) in obese subjects [6,7]. Besides the well documented beneficial effects of the high concentration of omega-3 polyunsaturated fatty acids (PUFA) found in various fish sources, many observations suggest that omega-3 PUFA are not the sole player involved in the beneficial properties of fish consumption on MetS. Indeed, a meta-analysis revealed that daily intake of as much as 3 g of fish oil alone has no beneficial effect on glycemia in T2D [8], while consumption of whole lean fish (24 g/day; providing only 140 mg of ω-3 fatty acid) was inversely correlated with the incidence of insulin resistance and T2D [9]. These latter findings are consistent with the hypothesis that a constituent other than lipid was responsible for the beneficial effect of fish consumption on glucose metabolism. In the last decade, studies carried out on animals and humans revealed that fish protein can improve insulin sensitivity and reduce obesity-linked inflammation [10,11,12,13,14,15]. More recently, it was found that a salmon protein hydrolysate reduces body fat and increases insulin sensitivity [16]. Protein hydrolysates from salmon and other fish sources (mackerel, bonito, and herring) were also found to reduce inflammation in visceral adipose tissue [16]. These observations are consistent with previous studies showing that dietary fish protein improves insulin sensitivity and reduces inflammation in insulin-resistant subjects [14,15]. Moreover, the fractionation of salmon protein hydrolysate suggested that low molecular weight peptides (<1 kDa) were highly responsible for those bioactivities [17]. However, the identification of these bioactive peptides (BPs) has never been accomplished. Consequently, BPs are suitable candidates for a new era of pharmaceutical products, especially with the heightened concerns of side effects of small molecule drugs [18]. Nevertheless, BPs are fragments that are encrypted in the primary sequences of proteins, conferring functions beyond basic nutritional benefit [19]. Because these peptides are generated by protein hydrolysis, they can represent only minor constituents in a highly complex matrix, further separation steps are needed to concentrate these peptides and optimize their bioactive effects [20]. 

Hence, very recently, the efficiency of electrodialysis with ultrafiltration membrane (EDUF) to generate bioactive fractions from a complex hydrolysate such as a salmon by-product hydrolysate, was demonstrated [21]. In fact, in this study, it was observed that anionic peptides concentrated in all three anionic recovery compartments (ARC1, ARC2 and ARC3) could increase the glucose uptake in basal condition. Moreover, it was also observed that the initial hydrolysate, when depleted in cationic peptides, also showed an increase of the glucose uptake, as well as the fraction in basal condition was able to mimic the insulin action and demonstrated the same efficiency to increase the glucose uptake than insulin control. Nevertheless, in the previous study by our team [21], the bioactive properties were focused only on the glucose uptake, whereas T2D is characterized by the defective insulin secretion from pancreatic beta cell resulting from an effort to compensate for various abnormalities such as hepatic insulin resistance and an impaired muscle glucose uptake associated to a chronic inflammatory state [22]. 

In this context, the study aimed to generate bioactive fractions against T2D and to identify peptide sequences responsible for these bioactivities. Hence, three bioactivities were studied, such as glucose uptake, hepatic glucose production (HGP) and anti-inflammatory properties. Furthermore, the most promising sequences were chemically synthetized and individually tested for their capacity to increase glucose uptake, to decrease HGP and to decrease inflammation. 

## 2. Materials and Methods

### 2.1. Materials and Electrodialysis Cell

#### 2.1.1. Hydrolysate Preparation

The salmon protein hydrolysate (SPH) was produced according to the procedure described previously by Chevrier et al. [17]. Briefly, deboned salmon frames were homogenized in a 1.0 M NaOH solution before isoelectric precipitation. Then, a sequential hy- drolysis was carried out with pepsin, then trypsin and chymotrypsin. The supernatant was filtered through a 5 µm pore size paper filter to remove any insoluble fat or protein and, afterwards, the filtrate was ultrafiltered using a Prep/Scale Tangential Flow Filtration (TFF) 2.5 ft^2^ cartridge with 1 kDa size-exclusion limit (Millipore Corporation, Bedford, MA, USA). The permeates containing peptides with molecular weights <1 kDa were collected, demineralized by electrodialysis and freeze-dried.

#### 2.1.2. Chemicals

Na_2_SO_4_ from Laboratoire MAT (Quebec City, QC, Canada), 1.0 M NaOH and HCl solutions as well as acetonitrile optima^®^ LC/MS were provided from Fisher Scientific (Montréal, QC, Canada). Trifluoroacetic acid (TFA) was obtained from J.T. Baker (Phillipsburg, NJ, USA) and KCl was purchased from ACP Inc (Montreal, QC, Canada). Fetal Bovine Serum (FBS) and trypsin (0.25% solution) were obtained from Invitrogen (Burlington, ON, Canada). Nylon filters were from Mandel Scientific (Guelph, ON, Canada). 2-déoxy-D-glucose (nonradioactive), CaCl_2_, Hepes-Na and MgSO_4_ were obtained from Sigma Aldrich (Oakville, ON, Canada), NaCl from VWR international (Montreal, QC, Canada). D-2-deoxy-[3H] glucose (radioactive) from Perkin Elmer (Woodbridge, ON, Canada). Finally, Pierce^®^ BCA Protein Assay Kit BCA, from Pierce Biotechnology (Rockford, IL, USA).

#### 2.1.3. Peptide Synthesis

Peptide synthesis and purification were carried out by the Laboratoire de chimie peptidique et biomoléculaire du CHU de Québec (Québec, QC, Canada). Peptides were synthesized by standard Fmoc solid-phase synthesis using 1-Cl-Trt resin [23,24]. A purity level of ≥90% for each sequence was achieved. Briefly, the Fmoc protecting group was removed from the resin by two 10 min treatments with 20% piperidine in dimethylformamide (DMF, *v*/*v*), and amino acid coupling was performed with Fmoc- Xaa-OH (3 equivalents), 1-(6-Chloro-1H-benzotriazole-1-yl)-1,1,3,3-tetramethylaminium hexafluorophosphate (HCTU, 3 equivalents) and N-methylmorpholine (12 equivalents) in dimethylformamide (DMF, 2 × 30 min). The synthesized peptides were released by treating the resin with 20% hexafluoro-1-propanol (HFIP) in dichloromethane (DCM) during 30 min [25]. Side chain deprotection was achieved by treating the peptides with TFA/triisopropylsilane (TIPS)/H_2_O (95:2.5:2.5, *v*/*v*/*v*) during 3 h prior to precipitation with cold ether and purification by RP-HPLC (Shimadzu Prominence instrument (Columbia, MD, USA)) using a Vydac 218 MS column (300 Å 22.0 × 250 mm, 10 μm, C18) with a 0.1% TFA/H_2_O (solvent A) and 0.1% TFA/CH_3_CN (solvent B) linear gradient of 10–100% solvent B for 20 min at a flow rate of 10 mL/min and UV detection at 220 nm and 254 nm. Freeze-dried purified peptides were characterized by a hybrid ion mobility quadrupole TOF mass spectrometer (6560 high-definition mass spectrometry (IM-Q-TOF), Agilent, Santa Clara, CA, USA). 

Before being tested in-vitro, the chemically synthesized peptides were simply diluted in HPLC grade water at a 1 mg/mL concentration and the mother solution was diluted with a specific volume of culture medium to obtain the final desired concentration to be tested and to be added with the cells. The control was the same volume of culture medium added to the cells.

#### 2.1.4. Membranes

For EDUF, one polyether sulfone (PES) ultrafiltration membrane with a molecular weight cut-off of 50 kDa was purchased from Synder filtration (Vacaville, CA, USA). Food grade Neosepta CMX-SB cationic membranes as well as Neosepta AMX-SB anionic membranes were obtained from Astom (Tokyo, Japan). 

#### 2.1.5. Electrodialysis Configurations

**Figure 1:**
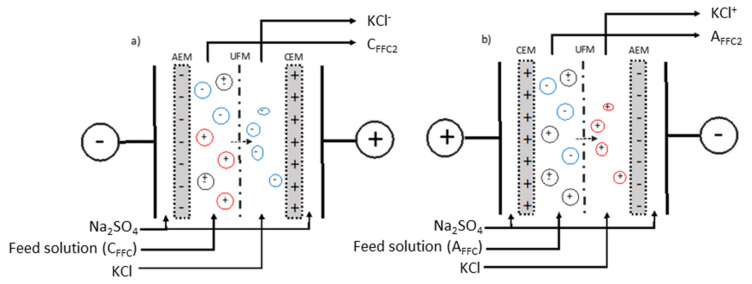
Schematic of electrodialysis with ultrafiltration membrane (EDUF) cells: (**a**) configuration 1—for the fractionation of C_FFC_ (Cationic final feed compartment), and (**b**) configuration 2—for the fractionation of A_FFC_ (Anionic final feed compartment), generated from a previous work [21].

The electrodialysis cell used for the experiments was an MP type cell with an effective surface area of 100 cm^2^, manufactured by ElectroCell Systems AB Company (Täby, Sweden). The cell was composed of one anion-exchange membrane (AEM), one cation-exchange membranes (CEM), one ultrafiltration membrane (UFM) with MWCO 50 kDa as illustrated in Figure 1 and as described in [21]. 

The first configuration—configuration 1 (Figure 1a) was arranged for the separation of anionic peptides. The cell was divided into three closed loops; one contained 1.5 L of a KCl solution (2 g/L) for the recovery and concentration of anionic peptides (KCl^−^). The feed solution consisting of the Cationic Final Feed Compartment (C_FFC_) generated from a previous EDUF separation [21] was circulated in the compartment between the UFM and AEM. The recovery solution from the feed compartment was called C_FFC2_. The last loop contained the electrode rinsing solution (20 g/L, Na_2_SO_4_, 3 L), split into two streams circulating into both electrolyte compartments.

In the second configuration—configuration 2 (Figure 1b), the compartment con- taining a KCl solution circulating between the UFM and AEM allowed the recuperation of cationic peptides (KCl^+^). The feed solution was circulated in the compartment between the UFM and CEM. The feed solution consisting of the Anionic Final Feed Compartment (A_FFC_) generated from a previous EDUF separation [21], and the final solution recovered in this compartment was called A_FFC2_. The rinsing electrode solution was circulated into both electrode compartments as for the anionic configuration.

#### 2.1.6. Electroseparation Protocol

The spray dried SPH was diluted with deionized water at a protein concentration of 0.7% (*w*/*v*) and the EDUF fractionation was performed for 4 h. EDUF experiments were carried out in batches for both cell configurations at a constant electrical field strength of 6 V/cm (corresponding to a current density varying between 0.005 and 0.008 A/cm^2^ during the treatment). The system temperature was maintained around 16 °C to prevent microbial growth [26]. The EDUF separations were done at pH 6, the same pH used in our previous study. Indeed, in that study, the final solution recovered in the feed compartment enhanced the glucose uptake at 1 ng and 1 μg/mL in both conditions (basal and stimulated by insulin), and the cationic peptides recovered in the Cationic Recovery Compartment (CRC) stimulated the bioactivity under the basal condition at both 1 ng and 1 μg/m [21]. From these results, the pH’s of SPH and recovery (KCl) solutions were adjusted to 6 before each run with 0.1 N NaOH and/or 0.1 N HCl and maintained constant thereafter [21]. For each treatment, volumes of 10 mL of SPH and recovery solutions were collected before applying voltage, and then every hour during the treatment to determine the peptide migration rate and their kinetics of migration. The electrical conductivity of the SPH feedstock and recovery solutions was kept constant by the addition of KCl, as seen in Suwal et al. [26]. The current intensity and electrical potential differences of the AEM, CEM and UFMs were recorded every 30 min during EDUF experiments for both configurations. Finally, for each condition, 3 replicates were carried out. A cleaning-in-place procedure was performed after each replicate, according to the membrane manufacturer’s instructions and the cell was dismantled before being reassembled.

### 2.2. In-Vitro Experiments and Analyses

#### 2.2.1. Glucose Uptake Experiments 

Glucose uptake experiments were performed as previously described by Henaux et al. [21], Roblet et al. [27] and Tremblay et al. [28]. Briefly, L6 skeletal muscle cells were grown in an α-minimum essential medium (α-MEM) containing 2% (*v*/*v*) fetal bovine serum (GBS) in an atmosphere of 5% CO_2_ at 37 °C. Cells were plated at 600,000 cells/plate in 24-well plates to obtain about 25,000 cells/mL. The cells were incubated 7 days, to reach their complete differentiation to myotubes (7 days post-plating). A volume of 10 µL of EDUF fractions has been tested at a concentration of 1 µg/mL and 1 ng/mL, and synthesized peptides at a concentration of 1 ng/mL. After experimental treatments, cells were rinsed once with 37 °C HEPES-buffered solution (20 mM HEPES, pH 7.4, 140 mM NaCl, 5 mM KCl, 2.5 mM MgSO_4_, and 1 mM CaCl_2_) and were subsequently incubated in HEPES-buffered solution containing 10 µM 2-deoxyglucose and 0.3 µCi/mL 2-deoxy-[^3^H] glucose for 8 min. The radioactivity was determined by scintillation counting. Protein concentrations were determined by the BCA method, and glucose uptake results were expressed in pmol/min.mg of protein. The experiments were repeated 6 times, and each repetition was run in triplicate.

#### 2.2.2. Hepatic Glucose Production Experiments

Hepatic glucose production experiments were performed as described by Chevrier et al. [17] with EDUF fractions or synthesized peptides at a concentration of 10 μL/well with or without insulin at 1 and 0.1 nmol, respectively. Briefly, FAO rat hepatocytes were grown and maintained in monolayer culture in Roswell Park Memorial Institute medium (RPMI) containing 10% FBS in an atmosphere of 5% CO_2_ at 37 °C. Cells were plated at 4 × 10^6^ cells/plate. FAO cells were deprived with 1 mL/well of RPMI without FBS, and the EDUF’s fractions were added at 10 μL/well with or without insulin at 1 nmol. Cells were washed three times with PBS, then incubated for 5h (in an atmosphere of 5% CO_2_ at 37 °C) with EDUF fractions at a concentration of 1 and 1 ng/mL, or with the synthesized peptides at a concentration of 1 ng/mL, in the presence or absence of insulin at 1 nmol in a hepatic glucose production medium. Glucose production was measured in the medium by using the Amplex Red Glucose/Glucose Oxidase Assay kit (Invitrogen, Waltham, MA, USA). Experiments were repeated 6 times, and each repetition was run in triplicate.

#### 2.2.3. Anti-Inflammatory Experiments

Anti-inflammatory experiments were conducted as described by Chevrier et al. [17]. Cells were plated at 4 × 10^6^ cells/plate for 24 h before the experiment, macrophages were stimulated with 2.5 ng/mL of lipopolysaccharide (LPS) in the presence of EDUF fractions at a concentration of 1 and 1 ng/mL, or synthesized peptides at 1 ng/mL for 16 h, and the accumulation of nitrite was used as an index of inducible NO synthase. Nitrite was measured by using the Griess method. Then, cells were lysed in 50 mmol NaOH, and protein content was determined by using BCA protein Assay kit. Experiments were repeated 6 times with the EDUF fractions and 3 times with the synthesized peptides, and each repetition was run in triplicate.

#### 2.2.4. Total Peptide Concentration in Dry Samples

The total nitrogen content was measured by the combustion of around 100 mg of sample powder (LECO-FP528 carbon nitrogen analyzer, LECO, St. Joseph, MI, USA) in order to obtain the final concentration of peptide in each peptide recovery compartment (KCl^+^, KCl^−^, A_FFC2_ and C_FFC2_) as well as in initial (C_FFC_ and A_FFC_) feed solutions. The nitrogen concentration obtained in the samples was converted into peptide percentage by multiplying with a conversion factor of 6.25, the value commonly used for crude fish proteins. 

#### 2.2.5. RP-UPLC and Mass Spectrometry Analyses

The RP-UPLC analyses were done according to the previous study from Henaux et al. [21] and Durand et al. [29] with a 1290 Infinity II UPLC (Agilent Technologies, Santa Clara, CA, USA) to separate samples before entering the mass spectrometer. All EDUF fractions were diluted at the same peptide concentration of 0.5 mg/mL (based on LECO total nitrogen determination) to allow comparison of the peptide area under the curve. Then the samples were filtered through 0.22 µm PVDF filter into a glass vial. A volume of 5 µL of each sample was loaded onto an Acquity UPLC CSH 130 1.7 µm C18 column (2.1 mm i.d. × 150 mm, Waters Corporation, Milford, MA, USA) at 400 µL/min and 45 °C. A linear gradient from 2% to 25% over 50 min and ramping to 90% until 57 min was used. The gradient was composed of solvent A (LC-MS grade water with 0.1% formic acid), and solvent B (LC-MS grade ACN with 0.1% formic acid). Each sample was run in triplicate for statistical evaluation of technical reproducibility.

A hybrid ion mobility quadrupole TOF mass spectrometer (6560 high-definition mass spectrometry (IM-Q-TOF), Agilent, Santa Clara, CA, USA) was used to identify the composition of each EDUF fraction. The method was identical as Henaux et al. [21]. The Salmonidae protein database was used to search and identified potential peptides.

#### 2.2.6. Statistical Analyses 

In-vitro bioactivity assays (Glucose uptake, hepatic glucose production and anti-inflammatory experiments) were subjected to a one-way analysis of variance (ANOVA) using SAS software version 9.1 (SAS institute Inc., Cary, NC, USA) with Dunnett’s post hoc test at a significant *p* value of 0.05 for acceptance. Peak areas of the chromatograms have been compared using a one-way analysis of variance (ANOVA) using SAS software version 9.1 (SAS institute Inc., Cary, NC, USA) with Tukey’s post hoc tests at a significant *p* value of 0.05 for acceptance.

## 3. Results and Discussion

### 3.1. Effects of EDUF Fractions 

#### 3.1.1. Glucose Uptake

**Figure 2:**
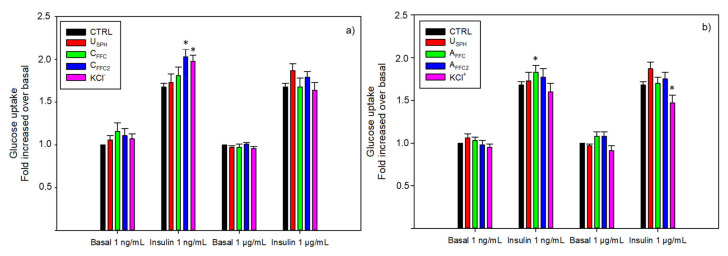
Glucose uptake modulation in L6 skeletal muscle cells with or without insulin stimulation by (**a**) the recovery compartments from EDUF configuration 1 and (**b**) the recovery compartments from EDUF configuration 2. Data represent mean ± SEM, *n* = 9. An asterisk indicates that mean values are significantly different (*p* < 0.05) from the control’s mean value.

Glucose intolerance has been identified as a major metabolic abnormality involved in the metabolic syndrome and a diabetes precursor, resulting in a deficiency of insulin to mediate muscle glucose uptake and to inhibit hepatic glucose production [30]. In-vitro experiments on L6 myocytes were performed to investigate the glucoregulatory effect of EDUF fractions. As presented in Figure 2, no effect was observed in any of the fractions under the basal condition. However, some EDUF fractions were able to promote insulin activity on glucose uptake. As shown in Figure 2a, both C_FFC2_ and KCl^−^ have significantly increased glucose uptake in the presence of insulin stimulation at 1 ng/mL of 21% and 15%, respectively, while the initial fraction, C_FFC_, showed no effect on the bioactivity. C_FFC2_ is deprived in anionic peptides, while C_FFC_ contains all anionic peptides.

Concerning the EDUF configuration 2, only the initial fraction (A_FFC_) demonstrated an enhancement (12%) of glucose uptake in presence of insulin stimulation (Figure 2b). The final fraction (A_FFC2_) showed no effect, and the recovered fraction, KCl^+^, concentrated in cationic peptides, had significantly decreased glucose uptake at 1 μg/mL in presence of insulin stimulation. Thus, these results suggested a potential antagonistic effect of down-regulation between peptides and inhibitory peptides which was canceled when peptides were separated into KCl^+^ and A_FFC2_ fractions.

Nevertheless, because bioactive peptides are generated by protein hydrolysis, they can represent only minor constituents in a highly complex matrix, and the bioactivity could be altered due to inhibition or interaction among peptides [31]. The use of subsequent separation steps is needed to enrich active peptide fractions from the hydrolysates [32]. For example, Roblet et al. [33] managed to generate bioactive anionic and cationic fractions, from a non-bioactive hydrolysate, depending on the pH used for the separation (3, 6 or 9). However, at pH 6 (pH used in this study) they obtained a cationic fraction able to increase glucose uptake, while our cationic fraction KCl^+^ showed a decrease. These different results can be explained by the different UFMs MWCO used for the separation (20 kDa instead of 50 kDa in our study), generating fractions of different composition in terms of size and sequences [34,35]. 

Furthermore, it was demonstrated, in our previous study, that the anionic peptides from a salmon by-product hydrolysate were responsible for the improvement of the glucose uptake in L6 muscle cells, while cationic peptides may be inhibitors of this bioactivity [21]. Indeed, from our first simultaneous EDUF separation it was concluded that anionic peptides may be involved in the glucose uptake response as anionic peptides recovered in A_RC1_ (Anionic Recovery Compartment 1), A_RC2_ (Anionic Recovery Compartment 2) and A_RC3_ (Anionic Recovery Compartment 3) demonstrated a significant increase in glucose uptake at both 1 ng/mL and 1 μg/mL under basal conditions. On the other hand, Cationic Recovery Compartment 3 (C_RC3_) from our previous study had decreased the bioactivity [21]. 

In addition to peptides, free cationic amino acids could have reached the KCl^+^ and decreased the bioactivity. It was previously demonstrated that free cationic amino acids, generated by the enzymatic hydrolysis of various marine by-products, for example snow crab by-products [36] or herring milt [29], could be concentrated in recovery compartments. Moreover, some of these cationic amino acids, such as histidine, cysteine and tyrosine have been identified as inhibitors of glucose uptake in presence of insulin stimulation [32]. The authors concluded that these amino acids may potentiate the activation of the mTOR/p70S6k pathway, that negatively modulates the ability of insulin to transmit signals to PI 3- kinase via IRS-1 and leading to an insulin resistance [28].

#### 3.1.2. Hepatic Glucose Production 

**Figure 3:**
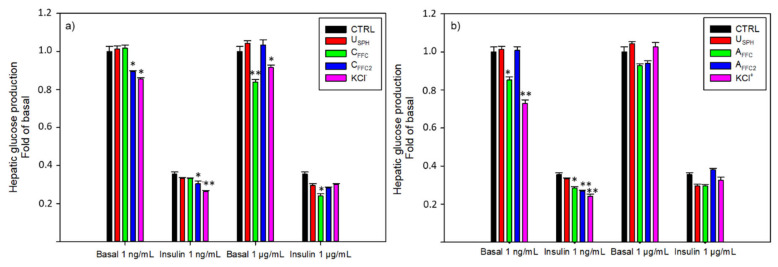
In-vitro hepatic production from FAO cells with or without insulin inhibition by (**a**) the recovery compartments from EDUF configuration 1 and (**b**) the recovery compartments from EDUF configuration 2. Data represent mean ± SEM, *n* = 6. An asterisk indicates that mean values are significantly different (*p* < 0.05) from the control’s mean value.

The capacity of the EDUF fractions to modulate basal and insulin hepatic glucose production (HGP) in FAO cells was investigated and results are presented in Figure 3. Once more, no effect was observed concerning the USPH. Nevertheless, as presented in Figure 3a, KCl^−^ fraction decreased the HGP in basal condition of 17 and 9% at 1 ng/mL and 1 μg/mL, respectively. These anionic peptides recovered in the KCl^−^ fraction have also improved the insulin effect on the HGP by 35% at 1 ng/mL compared to the insulin control. Finally, both fractions recovered in the feed compartments (C_FFC_ and C_FFC2_) were bioactive at both concentrations: C_FFC_ decreased the HGP at the highest concentration tested (1 μg/mL) in basal (11%) and insulin (14%) conditions, whereas C_FFC2_ decreased HGP at the lowest concentration tested (1 ng/mL), also in basal (16%) and insulin (34%) conditions. Concerning the EDUF configuration 2 (Figure 3b), cationic peptides recovered in the KCl^+^ fraction demonstrated the highest bioactivity in basal (28%) and insulin (36%) conditions at 1 ng/mL, compared to control conditions. Moreover, A_FFC_ also demonstrated a decrease in HGP (17 and 25%) at 1 ng/mL (without and with insulin stimulation, respectively). Finally, A_FFC2_ decreased the HGP by 25% at 1 ng/mL comparatively to the insulin control. 

For the first time, EDUF fractions demonstrated the capacity to decrease the HGP in in-vitro models. Indeed, the use of subsequent separation steps increased the concentration of bioactive peptides in fractions and generated bioactive fractions. Interestingly, both recovery compartment fractions, KCl^−^ and KCl^+^, have shown the highest inhibition of the HGP compared to fractions from the feed compartment (C_FFC_ and C_FFC2_; A_FFC_ and A_FFC2_, respectively). Thus, charged peptides, able to cross the 50 kDa UFM, were involved in the reduction of the hepatic glucose production. As the migration was not complete, peptides concentrated in the recovery compartments might still be present in the feed compartments, a single peptide or a group of peptides found in all fractions could induce this HGP decrease.

#### 3.1.3. Inflammation

**Figure 4:**
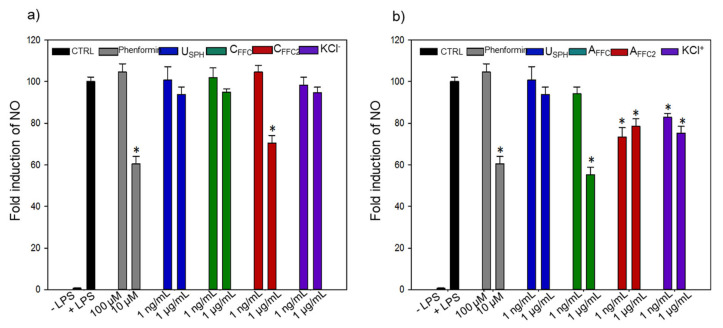
Inflammatory activity on J774 macrophages in absence or presence of LPS stimulation by (**a**) the recovery compartments from EDUF configuration 1 and (**b**) the recovery compartments from EDUF configuration 2. Data represent mean ± SEM, *n* = 6. An asterisk indicates that mean values are significantly different (*p* < 0.05) from the control’s (+LPS) mean value.

In-vitro anti-inflammatory effects of EDUF fractions were investigated and results are presented in Figure 4. All fractions were tested in the presence of LPS, to observe a possible reduction of the LPS-induced inflammatory effect measured by nitrite accumulation as an index of NO production. Moreover, phenformin, an antidiabetic drug used for the treatment of T2D, was used as a positive control to compare the effect of EDUF fractions. As for previous bioactivities tested, the USPH did not show any effect on the inflammation (Figure 4). However, C_FFC2_, from EDUF configuration 1 decreased LPS-induced iNOS activation in macrophages by 30% at 1 μg/mL (Figure 4a). Concerning configuration 2 (Figure 4b), A_FFC_ decreased the inflammation at 1 μg/mL. While A_FFC2_ (22 and 27%) and KCl^+^ (25 and 18%) have demonstrated a significant decrease in LPS-induced inflammation for both concentrations (1 ng/mL and 1 μg/mL, respectively). Interestingly, A_FFC__,_ at a concentration of 1 μg/mL, yielded 45% of reduction in inflammation, showing the same effect as the phenformin at 10 μM (40%) (*p*= 0.558). These results demonstrated the in-vitro reduction in LPS-induced iNOS activation. The removal of some cationic peptides from the initial feed (A_FFC_) decreased the anti-inflammatory effect. Indeed, a decrease of the inflammation was observed in A_FFC2_ compared to A_FFC_. However, as the experiments were carried out at the same concentrations (1 ng and 1 ug/mL of peptides), the relative concentration of cationic peptides in KCl^+^ was higher compared to A_FFC_ and A_FFC2_. Nevertheless, as previously explained, A_FFC_ had a better activity than the KCl^+^. Thus, a synergistic effect might exist between cationic peptides and other peptides in A_FFC_, resulting in a stronger anti-inflammatory effect than the one obtained for the isolated KCl^+^ fraction.

### 3.2. Peptide Sequence Identification and In-Vitro Experiments of Individual Synthesized Peptides

#### 3.2.1. Peptide Sequence Identification

All these results suggested that anionic peptides have gluco-regulatory effects by enhancing the glucose uptake in L6 muscle cells and decreasing the glucose production in hepatic cells, while cationic peptides decreased the inflammation in activated macrophages. In order to identify potential bioactive peptides, both fractions were characterized by MS/MS analysis coupled with a database for identification. Fifty-three compounds were found to be common to all these fractions, and 24 potential sequences were successfully identified by database comparison (Salmonidae database downloaded from [37]). Amongst the 24 peptide potential sequences identified, 13 peptides were chosen based on their scores obtained by comparison of their experimental MS/MS spectra and their theoretical MS/MS spectra to be chemically synthesized. The sequences of the 13 peptides were listed in Table 1. Each synthesized peptide was tested individually for its in-vitro activities on glucose uptake, hepatic glucose production and anti-inflammatory.

#### 3.2.2. Glucose Uptake

**Figure 5:**
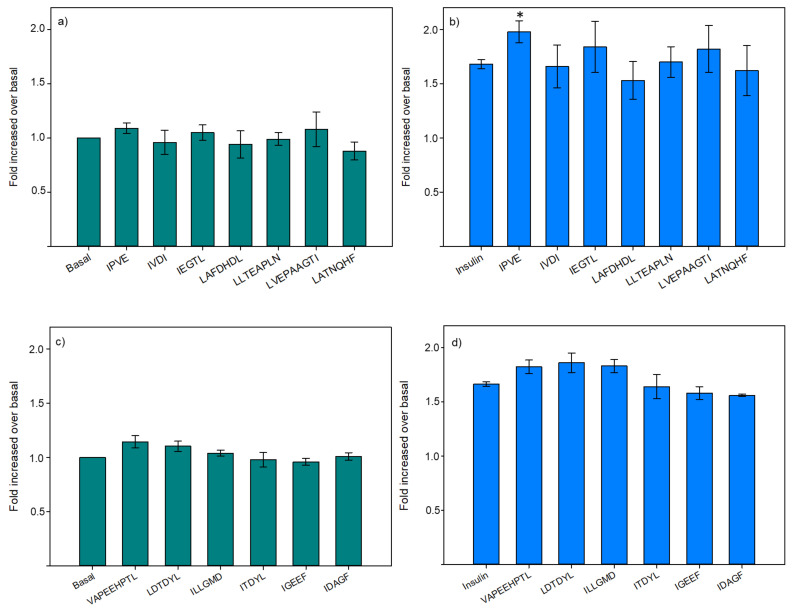
Effects of synthesized peptides on glucose uptake modulation in L6 skeletal muscle cells in basal (**a**,**c**) or insulin-stimulated (**b**,**d**) conditions. Data are expressed as mean ± SEM, *n* = 9. An asterisk indicates that mean values are significantly different (*p* < 0.05) from the control’s mean value.

For glucose uptake, all peptides were tested under basal conditions (Figure 5a,c) and in the presence of insulin (Figure 5b,d). No differences were observed in the basal condition whatever the peptide tested. While in the presence of insulin, IPVE demonstrated a significant enhancement of glucose uptake (17%) compared to the insulin control (*p* = 0.016). 

**Figure 6:**
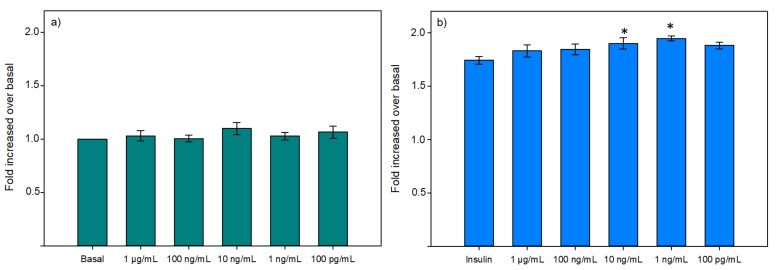
Dose-response effect of IPVE on glucose uptake modulation in L6 skeletal muscle cells in basal (**a**) or insulin-stimulated (**b**) conditions. Data are expressed as mean ± SEM, *n* = 9. An asterisk indicates that mean values are significantly different (*p* < 0.05) from the control’s mean value.

Afterward, the dose-response effect to IPVE was tested (Figure 6) with concentrations from 1 μg/mL to 10 pg/mL. These results confirmed the capacity of IPVE to improve glucose uptake in muscle cells, as it was able to increase the bioactivity at 10 ng/mL and 1 ng/mL. Also, this result showed that the response of IPVE was dose-dependent, as for the highest (>10 ng/mL) and the lowest (<100 pg/mL) concentrations tested, IPVE presented no effect on glucose uptake. Although not spectacular, the effect of IPVE concentration is nonetheless constant and significant. It is also important to keep in mind that in the pre-sence of insulin, the glucose transport is already highly stimulated in the muscle cell and that it can be then difficult to stimulate further the entry of glucose in a very marked way.

#### 3.2.3. Hepatic Glucose Production

**Figure 7:**
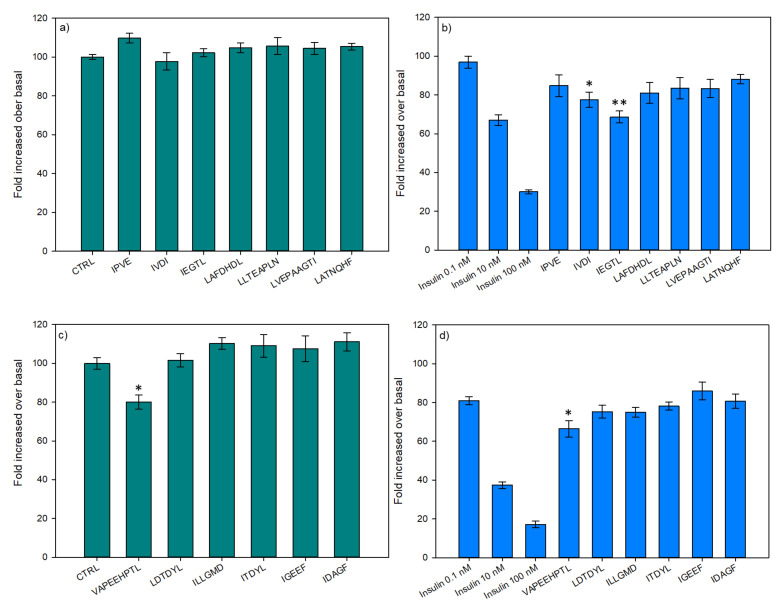
Effects of synthesized peptides on in-vitro hepatic production from FAO cells in basal (**a**,**c**) and insulin-stimulated (**b**,**d**) conditions, all at 1 ng/mL. Data are expressed as mean ± SEM, *n* = 6. An asterisk indicates that mean values are significantly different (*p* < 0.05) from the control’s mean value. Two asterisks indicate that mean values are significantly different (*p* < 0.01) from the control’s mean value.

For their capacity to decrease the HGP, all 13 peptides were tested in basal (Figure 7a,c) and insulin (Figure 7b,d) conditions. Concerning the first seven peptides (Figure 7a,b), results demonstrated no differences when tested under basal condition (Figure 7a). For the insulin condition, as the peptides were incubated with insulin at 0.1 nmol, the statistical comparisons were performed with the insulin control at 0.1 nmol. Two peptides demonstrated a decrease in their bioactivity when incubated with insulin. Indeed, IVDI and IEGTL have downregulated the HGP by 20% and 30%, when compared to insulin at 0.1 nmol, respectively. Moreover, IEGTL incubated with insulin at 0.1 nmol has shown the same capacity to decrease the HGP than insulin alone at 10 nmol. From the six remaining peptides (Figure 7c,d), VAPEEHPTL demonstrated a decrease of the HGP in basal (20%) and insulin-stimulated (18%) conditions.

#### 3.2.4. Inflammation

**Figure 8:**
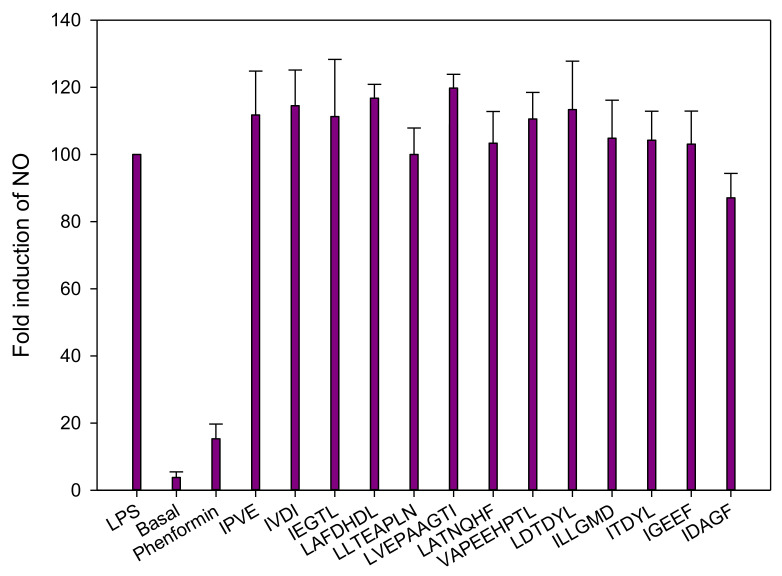
Effects of peptides on the nitric oxide (NO) production on lipopolysaccharide (LPS)-induced J774 macrophage cells. Data are expressed as mean ± standard deviation, *n* = 4.

All peptides were tested in the presence of LPS, to observe a possible reduction of the LPS-induced inflammatory effect. Moreover, phenformin, a commonly used antidiabetic drug for the treatment of T2D, was used to compare the effect of the peptides. According to these results, none of the peptides demonstrated a significant decrease of the NO production (Figure 8). However, as some fractions demonstrated a strong anti-inflammatory effect, the previous effect observed could be due to other peptides such as cationic peptides or due to an antagonistic effect among peptides. Recently, two new cationic peptide sequences (IVPAS and FDKPVSPLL) from herring milt hydrolysate were identified and demonstrated an in-vitro anti-inflammatory activity [29]. What is interesting here is that FDKPVSLL was present and identified in the A_FFC_ and C_FFC2_ fractions and might explain their anti-inflammatory activity. 

Numerous studies reported in the literature demonstrated the beneficial effects of salmon proteins and hydrolysates on T2D, however, none of these studies were able to identify the peptide sequences responsible for these beneficial effects. Furthermore, as these peptides sequences are new concerning the bioactivities tested in the present study, no information is available in the literature. However, what can be observed, is that the identified peptides in this study are mostly composed of hydrophobic amino acids such as Ala (A), Gly (G), Ile (I), Leu (L), Pro (P) and Val (V). It was previously described that antidiabetic and anti-obesity peptides are generally hydrophobic [39]. A mixture of hydrophobic amino acids, mostly composed of Ile (2 mM), but also by Cys (0.012 mM), Met (0.006 mM), Val (0.0016 mM) and Leu (0.014 mM) demonstrated the capacity to increase, in basal and insulin-stimulated conditions, glucose uptake in isolated rat epitrochlearis muscle [40]. These effects appear to be mediated via a pathway that is independent from the usual insulin cascade and, therefore, may prove effective as an alternative therapeutic treatment for the insulin resistance [40]. Indeed, as reported by Mîinea et al. [41], insulin increases the AKT phosphorylation, which inactivates AS160 by its phosphorylation and then promotes GLUT4 translocation [41]. However, the AA mixture seemed to increase the AS160 phosphorylation, thus promoting GLUT4 translocation, independently of the AKT phosphorylation. In another study, three hydrophobic peptides (IAVPGEVA, IAVPTGVA and LPYP) identified from soy glycinin, were able to increase the glucose uptake in HepG2 cells in another way. In that case, the glucose uptake was increased via GLUT1 and GLUT4 activation, through the adenosine monophosphate-activated protein kinase (AMPK) signaling pathways via phosphorylation of AKT and AMPK [42]. Moreover, the phosphorylation of AMPK, as reported by Mîinea et al. [41], can inhibit the mTOR/p70S6K pathway; its inactivation was described as a novel modulator of insulin-stimulated glucose transport in skeletal muscle cells [28]. Interestingly, these three peptides have the same antidiabetic molecular mechanisms as drugs like metformin [43] and thiazolidinediones [42,44]. In addition, leucine has also demonstrated its glucose metabolism effects by stimulating the glycogen synthesis via the inactivation of glycogen synthase kinase-3 in L6 cells [45]. Finally, it was reported that dipeptides mostly composed by hydrophobic AAs, such as IV, LV, VL, II, LI, IL and LL, were able to increase glucose uptake in muscle cells via the PI3-kinase and PKC (protein kinase C) pathways [46]. It has also been observed that the PI 3-kinase–dependent PKC activation was diminished in muscle and liver during insulin resistance and T2D [47]. Nevertheless, to better understand, how IPVE, IVDI, IEGTL and VAPEEHPTL, promote glucose metabolism, further analyses must be carried out on the molecular mechanisms of these BPs. It could be interesting to test different molecular pathways such as AKT/AS160 or the AKT/AMPK pathways, previously described. 

Moreover, another critical aspect in the development of BPs as natural new therapeutic approaches is their bioavailability. The bioavailability of peptides is the quantity that passes through the mucosal membranes in the intestine and stays available for action within the cells [48]. This bioavailability is generally affected by their physicochemical properties such as molecular size, charge, sequence, and solubility [39,49]. Thus, this bioavailability is determined by their sensitivity to the digestive enzyme peptidase and intestinal absorption. In humans, the gastrointestinal digestion of peptides starts in the stomach by the action of pepsin, and it continues in the luminal phase of the small intestine by the action of pancreatic proteases trypsin, α-chymotrypsin, elastase, and carboxypeptidase A and B [50]. These four BPs, as well as the EDUF fractions containing these peptides, were obtained by successive enzymatic digestion using three gastrointestinal enzymes: pepsin, trypsin, and chymotrypsin, and should not be affected by these gastrointestinal enzymes anymore. Nevertheless, if smaller peptides (di- or tri-peptides) can be transported across the enterocytes through intestinal-expressed peptide transporters (PEP1 and PEP2) [50], the transport of these oligopeptides through the intestine must be investigated. It was previously described that oligopeptides may be absorbed by passive transport through hydrophobic regions (transcellular transport) of membrane epithelia or tight junctions (paracellular transport) [51,52,53]. Additionally, hydrophobic peptides showed a higher permeation through biological membranes [39,50].

## 4. Conclusions

To the best of our knowledge, this is the first time that (1) peptide fractions generated after separation by EDUF were demonstrated as bioactive on three different bioactivities, all involved in the T2D, and (2) potential sequences involved in the improvement of glucose uptake and/or in the regulation of hepatic glucose production were identified from salmon by-product hydrolysates. Hence, two fractions were particularly promising: A_FFC_ and C_FFC2_ since they were both able to (1) increase glucose uptake in L6 muscle cells in the presence of insulin, (2) decrease hepatic glucose production in hepatic cells in both basal and insulin conditions and (3) decrease LPS-induced inflammation in macrophages. Moreover, A_FFC_, has shown an impressive capacity to decrease inflammation with a more important decrease than phenformin, a drug usually used to treat T2D. It also appeared from these results that anionic peptides concentrated in the KCl^−^ were able to increase glucose uptake and decrease HGP, while cationic peptides concentrated in KCl^+^ and present in both A_FFC_ and A_FFC2_ have shown good anti-inflammatory properties. Nevertheless, it is more probable that these bioactivities result from a synergistic effect among peptides than the effect of just one peptide. Finally, four new BP sequences were identified (IPVE, IVDI, IEGTL, and VAPEEHPTL), and their glucoregulatory effects were demonstrated. Nevertheless, the molecular mechanisms and their bioavailability must be determined before being considered natural new therapeutic agents for treating T2D. 

## Figures and Tables

**Table 1:** Sequences and characteristics of synthesized peptides.

Sequences Identified	Molecular Weight (Da)	Retention Time (min)	Net Charge (at pH 6)	pI	Scores *	% SPI **
IPVE	456.2581	10.174	-	4.60	7.97	73.1
IVDI	458.2738	22.342	-	3.80	6.6	78
IEGTL	531.2890	17.270	-	4.00	9.93	77.3
LAFDHDL	829.3972	24.588	-	4.19	10.19	79.3
LATNQHF	829.3972	24.588	+	6.74	11.26	80,8
LVEPAAGTI	869.4857	18.700	-	3.99	7.74	77.9
LLTEAPLN	869.4857	18.700	-	3.99	8.93	79.2
VAPEEHPTL	991.4970	15.796	-	4.50	17.53	93.7
LDTDYL	738.3424	40.044	-	3.56	8.26	81.3
ILLGMD	660.3504	29.454	-	4.30	10.63	81.3
ITDYL	623.3160	25.851	-	3.80	11.59	89.1
IGEEF	593.2770	20.050	-	3.79	9.05	77.8
IDAGF	521.2483	27.307	-	3.79	7.83	81.7

* Score of an individual peptide. It reflects the information content in the MS/MS spectrum [38]. >15 (quality: outstanding) and combined with % SPI of 60 or greater, very likely to be valid. >9 (quality: good) and combined with % SPI of 60 or greater, likely to be valid. >5 (quality: mixed quality) and combined with a % SPI of 60 or greater, review results to determine whether interpretation is valid. ** % SPI: percentage of the extracted MS/MS ion current explained by theoretical fragmentation of the database hit [38].

## Data Availability

Data is contained within the article.
